# Manual Therapy of Dysphagia in a Patient with Amyotrophic Lateral Sclerosis: A Case Report

**DOI:** 10.3390/medicina60060845

**Published:** 2024-05-22

**Authors:** Ilaria De Marchi, Francesca Buffone, Alessandro Mauro, Irene Bruini, Luca Vismara

**Affiliations:** 1 Department of Neurology and ALS Centre, Traslational Medicine, University of Piemonte Orientale, Maggiore della Carità Hospital, 28100 Novara, Italy; ilaria.demarchi@hotmail.it; 2 Division of Paediatric, Manima Non-Profit Organization Social Assistance and Healthcare, 20125 Milan, Italy; francescabuffone.ost@gmail.com; 3 Research Department, SOMA Istituto Osteopatia Milano, 20126 Milan, Italy; 4 Principles and Practice of Clinical Research (PPCR), Harvard T.H. Chan School of Public Health–ECPE, Boston, MA 02115, USA; 5 Division of Neurology and Neurorehabilitation, IRCCS Istituto Auxologico Italiano, Strada Luigi Cadorna 90, 28824 Piancavallo-Verbania, Italy; alessandro.mauro@unito.it (A.M.); lucavisma@hotmail.com (L.V.); 6 Department of Neurosciences “Rita Levi Montalcini”, University of Turin, 10126 Turin, Italy

**Keywords:** osteopathic medicine, amyotrophic lateral sclerosis (ALS), dysphagia, central nervous system, osteopathic manipulative treatment (OMT)

## Abstract

Amyotrophic lateral sclerosis (ALS) is an incurable rare neurodegenerative condition, with 45% of cases showing the symptom of dysphagia; its clinical signs are atrophy, weakness, and fasciculations of the facial muscles, tongue, and pharynx. Furthermore, dysphagia is the main cause of aspiration pneumonia. The traditional treatment for dysphagia varies based on the patient’s difficulty of swallowing. The initial phase consists of dietary consistency adjustments, progressing to alternatives like nasogastric tubes or percutaneous endoscopic gastrostomy (PEG) in advanced stages. Osteopathic manipulative treatment (OMT) is a complementary ‘hands-on’ approach that has already shown positive results as an add-on therapy in various health conditions. This study is a case report of a man diagnosed with ALS with initial dysphagia, managed with a protocol that extraordinarily included OMT. The patient showed somatic dysfunctions in the mediastinal region, upper cervical region, and occipital area which are all anatomically related to the nervous system, especially the glossopharyngeal reflex. At the end of the rehabilitation protocol, there was a reduction in the swallowing problems measured with Strand Scale and swallowing tests, and the patient reported an improved psycho-physical well-being assessed with the Amyotrophic Lateral Sclerosis Assessment Questionnaire (ALSAQ-40). Instead, the neurological function measured with ALSFRS-S remained stable. Although the nature of this study design prevents any causal assumption, the positive results should lead to future randomized controlled trials to assess the effectiveness of OMT as an adjunctive therapeutic proposal to improve the health of ALS patients.

## 1. Introduction

Amyotrophic lateral sclerosis (ALS) is an incurable degenerative disease of the central nervous system, involving the upper and lower motor neurons in the cerebral cortex, brain stem, and anterior horns of the spinal cord. Over the years, a heterogeneous distribution has been identified among different areas of the world. The average incidence of ALS worldwide is approximately 1.68 cases/per year per 100,000 inhabitants. The prevalence is about 10–12 cases per 100,000 inhabitants, and individuals of all populations and ethnic groups are affected, even if a higher incidence is noted in the Caucasian ethnic group. The incidence and prevalence increase after the age of 40, with a peak between 58 and 63 years [1,2,3]. As this is an incurable disease, the approved drugs can only slow it down, and researchers continue to study other possible treatments for both the management and cure of ALS.

In addition to the already challenging condition of ALS, 45% of cases show dysphagia, particularly in the bulbar forms. It manifests as atrophy, weakness, and fasciculations of the facial muscles, tongue, and pharynx; it is the most important cause of protein–calorie malnutrition and is the leading cause of aspiration pneumonia [4,5]. The guidelines on the management of an adult dysphagic patient recommend some functional assessments of the patient: morphodynamic assessment of the structures involved in swallowing that can be explored directly (lips, tongue, hard palate, veil of the palate, mandible, larynx and muscle control of the head); evaluation of buccofacial practices; evaluation of the superficial and deep sensitivity of the peri-buccal skin, lips, tongue, and palate; evaluation of cough, vomiting, and swallowing reflexes; evaluation of the presence of pathological reflexes; and, finally, swallowing tests with substances and foods of different consistencies. The instrumental assessment is only conducted when there is a suspicion of inhalation during the functional assessment [6,7,8]. The gold standard for the treatment of late-stage dysphagia, where there is also a significant loss of body weight, is the choice of alternative feeding methods, including the use of a nasogastric tube or percutaneous endoscopic gastrostomy (PEG). Both of these methods can lead to various complications, including pressure ulcers, esophagitis, rhinitis, aspiration pneumonia, and frequent replacement by dislocation or occlusion [9].

Recent studies have started to assess the possible contribution and effect of osteopathic manipulative treatment (OMT) in various health conditions, as well as in the neurological field [10,11,12]. OMT is a ‘hands-on’ approach that treats various health conditions following the “structure–function” relationship, in accordance with the different described models: biomechanical, neurological, respiratory/circulatory, biopsychosocial, and metabolic/bioenergetic [13,14]. Its assessment and treatment are performed according to the so-called somatic dysfunction, an altered function of the body that can manifest in different areas [15,16].

In this case report, we describe the clinical case of a man diagnosed with ALS with initial dysphagia who underwent an 8-week OMT protocol in addition to the standard care, assessing neurological, logopedic, and psychological functions as well as any adverse events.

### Detailed Case Presentation

The protocol for this study was approved by the local Ethical Committee (CE 179/2022) and was conducted in strict accordance with the Declaration of Helsinki. Before enrolling, the patient signed an informed consent form. This case report was reported according to the CARE Checklist.

The patient under examination was a 51-year-old Italian male, a commercial agent, not exposed to toxic agents. The first symptoms (progressive dysarthria and liquid dysphagia) began in June 2021. He came to the Amyotrophic Lateral Sclerosis Regional Expert Centre (CRESLA) of the “Maggiore della Carità” University Hospital (Novara, Italy) in February 2022. The first general neurological evaluation showed dysarthria, normal tongue with initial atrophy of the edges, and rare fasciculations. The masseter and peri-buccal reflexes were accentuated. At the spinal level, there was an initial hypotrophy with fasciculations at the right femoral quadriceps. The deep tendon reflexes were markedly accentuated in all locations. Nothing relevant was present in the previous anamnesis; the patient reported only a fall with a consequent slight head trauma during adolescence. He also denied a family history of neurodegenerative diseases.

For the diagnosis, various diagnostic tests were performed, including a brain MRI which documents bilateral frontotemporal atrophy and an electromyographic examination with diffuse active denervation in cranial, cervical, and lumbar districts. In March 2022, a genetic examination of the main involved genes in ALS (SOD1, FUS, TARDBP, C9orf72) was carried out, showing a negative result. At the first logopedic evaluation, the Strand Scale of signs and symptoms of dysphagia [17] was administered, showing slowed masticatory movements during the oral preparation and pharyngeal phases; in addition, the triggering of the pharyngeal reflex was delayed with a slight tendency to the pre-swallowing fall of the liquid bolus. According to the guidelines for dysphagia [6,7,8], for this patient, no instrumental tests were performed as they were not deemed essential at the time of enrollment. For the second neurological evaluation, the Revised Amyotrophic Lateral Sclerosis Functional Rating (ALSFRS-R) scale was used [18], showing a score of 45 out of 48. Because of the feeling of stiffness in the jaw reported by the patient and the mild sialorrhea, the neurologist prescribed amitriptyline. Finally, for the psychological evaluation, the Amyotrophic Lateral Sclerosis Assessment Questionnaire (ALSAQ-40) was administered.

In addition to the standard routine, the patient underwent an osteopathic evaluation and a weekly 45 min OMT session for a period of 8 weeks performed by a physiotherapist with 6 years of experience and attending the last year of osteopathy. The evaluation involved the functional status of the musculoskeletal, visceral, dural, and fascial systems; it was performed according to the Variability Model, assessing the somatic dysfunction. It analyzes the joint motion variability around the joint rest position, defined as the neutral zone (NZ). The different body areas were treated according to the severity of the somatic dysfunction:

- Grade 0—No SD: symmetric motion;
- Grade 1—Mild SD: asymmetric motion;
- Grade 2—Moderate SD: asymmetric variability of motion in the NZ combined with altered tissue texture or tenderness;
- Grade 3—Severe SD: asymmetric variability of motion in the NZ combined with altered tissue texture + tenderness. [15,16]

**Figure 1:**
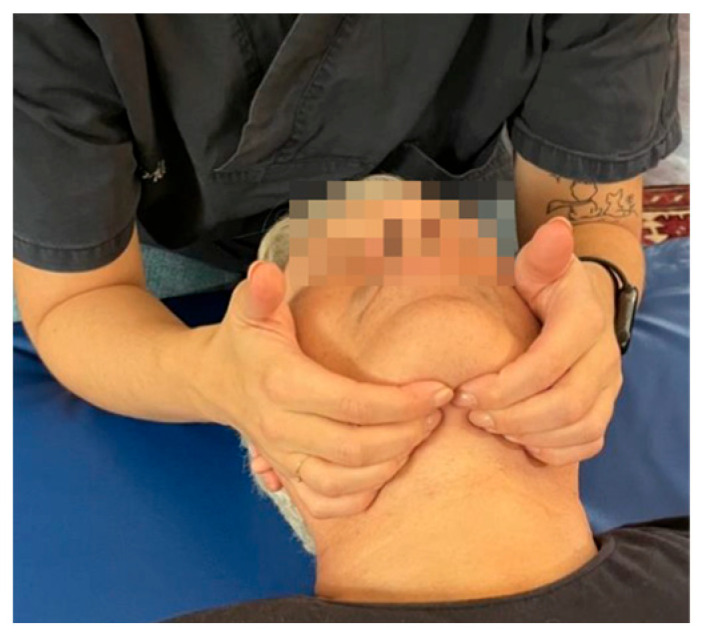
Buccal floor inhibition technique.

**Figure 2:**
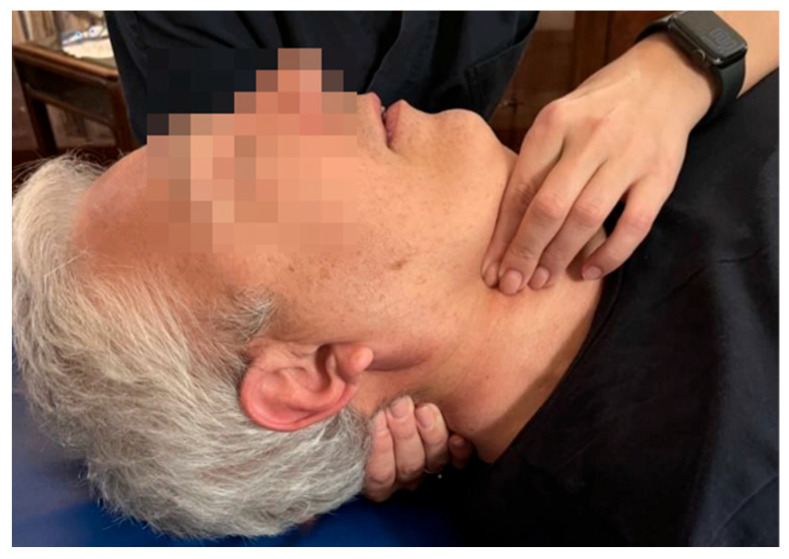
Anterior cervical arch technique.

**Figure 3:**
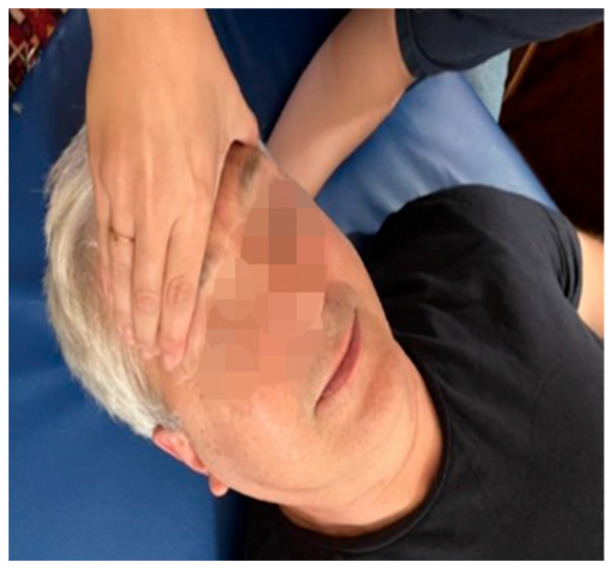
Decompression of the sphenobasilar synchondrosis technique.

**Figure 4:**
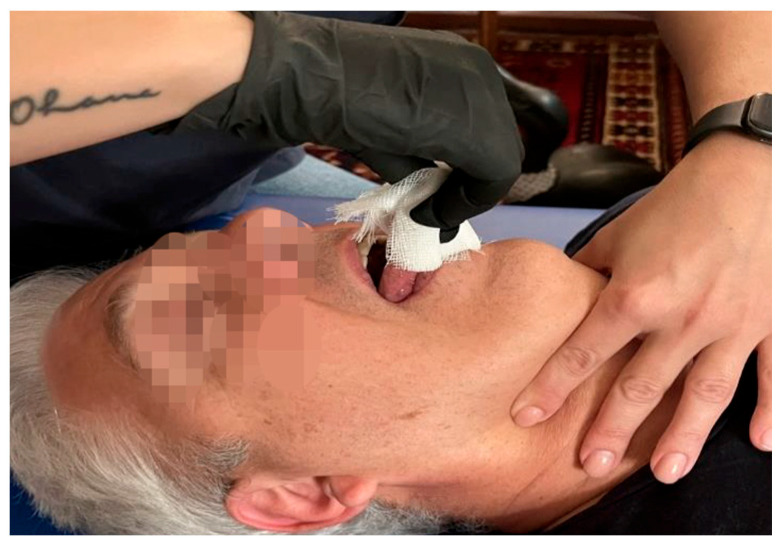
Tongue-structure fascial agreement technique.

According to this evaluation, the main somatic dysfunctions were at the following levels: at the atlanto-occipital joint, hyoid bone, cricoid cartilage, occipitomastoid suture, sphenobasilar synchondrosis, resulting in compression, and at the middle and deep cervical fascia in relation to the internal tissues of the rib cage, endothoracic fascia, and pleura (see Table 1 for further details). Therefore, along with the drug regimen, the somatic dysfunctions were treated with indirect manual techniques, which are characterized by a passive movement of the dysfunctional region into a position of less tissue tension [16]. The patient mainly received balanced treatment of buccal floor ligament tension, muscle inhibition treatment with craniosacral condylar decompression, and functional visceral system release treatment (see Table 2 and Figure 1, Figure 2, Figure 3 and Figure 4).

No adverse events occurred due to OMT. Three days after the fourth OMT session, the patient reported increased dysarthria salivation in the morning; however, it was due to the missed intake of the amitriptyline for a few days, and it resolved spontaneously.

In week 8, during the standard multidisciplinary visit, the patient repeated the logopedic, psychological, and neurological evaluations: the swallowing test showed an improvement for the masticatory movements, which were now suitable for solid foods; there was only a slight tendency to fall pre-swallowing with a liquid bolus taken in the free oral cavity. Also, ALSAQ-40 showed an improvement both in the patient’s perception of fatigue during meals and in the emotional component. Instead, the ALSFRS-S remained stable (see Table 3 for further details).

## 2. Discussion

ALS is a rare, inevitably fatal neurodegenerative disease with no therapies to date that can modify its course. Dysphagia is one of the most critical problems in patients with neuromuscular diseases and is related to increased morbidity and mortality [18]. Among the various factors that influence this problem are the form of onset of the disease, possible genetic mutations, age of onset, and rate of progression. Typically, there are some early signs related to dysphagia, including low voice, increased salivation, and weight loss. These disorders, related to muscle weakness, lead to serious complications, such as malnutrition, dehydration, and aspiration pneumonia (one of the main causes of respiratory crises and patient hospitalization) [19].

Early detection of these symptoms should contribute to better patient management and possible prevention of comorbidities, including the impact on quality of life. Although guidelines and previous studies considered fiberoptic endoscopic evaluation and videofluoroscopic swallowing as the gold standards for dysphagia in patients with and without neurodegenerative diseases [18,20,21,22], they are diagnostic tests and not treatments.

The traditional treatment of dysphagia varies according to the patient’s difficulty in swallowing, and the different phases of swallowing are evaluated using the Scale Strand [23]; according to the obtained result, different strategies are indicated during meals.

During the initial phase, the consistency of the diet is modified from a free diet to a soft or semi-liquid diet. Then, in the advanced stage of dysphagia, there are two alternative strategies to oral feeding: the nasogastric tube or the PEG. These are two methods that exclude feeding orally, but these interventions can in turn cause patient discomfort and related recurring problems, such as rhinitis, esophagitis, dislocations, decubitus lesions, and tube occlusions [24].

To date, there is only a pilot study using OMT as an adjunctive therapy for ALS patients, even if it did not address dysphagia and did not provide positive results. However, it is interesting to note that patients showed somatic dysfunctions at the cervico-dorsal levels more frequently than the healthy matched controls [10]. Similarly, in this clinical case, the patient had altered tissue tension in the mediastinal region, which is strictly related to neck structures and the base of the skull via the fascial layers [25], suggesting an unbalanced distribution of the myofascial load. Therefore, we believe that through the application of techniques on the cervical fascias, we can influence the functioning of the peri-laryngeal muscles, normalizing the sliding of the fascial layers and then removing the fascial tensions that could hinder the laryngeal movements, the proprioceptive component, thus increasing the perception of that area and the neuromuscular coordination; consequently, with osteopathic manipulative treatment we would have obtained an effect not only on the laryngeal movement excursion, but also on the fine coordination of the intrinsic muscles [26,27]. Furthermore, the upper cervical region and the occipital area—anatomically related to the nervous system, especially to the glossopharyngeal nerve, responsible for swallowing [13]—were also dysfunctional. The basioccipital gives insertion to the superior constrictor muscle of the pharynx; hypertonia of this muscle can affect the mobility of the atlo-occipital joint, as well as the tension of the pharyngeal resonance cavity and the mandibular function. In order to normalize this muscle, it is necessary to treat its proximal and distal insertions, that is, not only the base of the skull, but also the mandible and the tongue [28]. As the swallowing difficulty is caused by the inefficiency of oral transit, reduction in the movement of the tongue base, laryngeal elevation, and slowed gag reflex during the pharyngeal phase [29], addressing the somatic dysfunctions at these levels with OMT may have contributed to improving dysphagia in the short term.

A cure for ALS has not been found; nonetheless, its variety of symptoms can be supported with different therapies [29]. OMT may join the adjunctive interventions and complement the traditional treatment of dysphagia to slow down the progression and lengthen as much as possible the use of alternative strategies to oral feeding.

The osteopathic evaluation, analyzing the functional alteration of the movement [15,16], identified the areas of greatest dysfunctionality, and OMT, affecting the fascial and neurological components, improved the functionality of the described areas, improving the patient’s condition.

We are aware that the results obtained have a limitation as this is a case report, but the introduction of OMT in therapeutic management improved the patient’s dysphagia in the short term. This case underlines the importance, at a clinical level, of considering OMT as an adjuvant therapy to the traditional treatment of dysphagia and, at a scientific research level, of being a starting point for a randomized controlled trial with a large number of patients.

Since ALS is a multisystem disease, we hypothesize that OMT could be part of the therapeutic proposals to improve the health of the patient suffering from ALS. However, to date, there are not enough studies to allow the introduction of OMT in daily practice for ALS patients. This case report outlined an improvement in the ALSAQ-40, Strand Scale, and DYALS, suggesting that OMT is a safe adjunctive therapy for ALS with dysphagia; however, the nature of the design prevents any kind of causal assumption. We suggest the development of future randomized controlled trials with OMT in ALS patients with dysphagia to evaluate its possible effects in the medium and long term.

## Figures and Tables

**Table 1:** Summary of the osteopathic physical examination at the initial consultation.

Fascial system	Dysfunctions affecting the middle and deep cervical fascia
Visceral system	Esophageal and pharyngeal dysfunctions
Craniosacral system	Compressed sphenobasilar synchondrosis Occiput-mastoid suture dysfunction
Musculoskeletal system	Atlo-occipital joint dysfunction Posterior hyoid bone dysfunction

**Table 2:** Patient osteopathic manipulation treatment program over 8 weeks.

STAGE	GOALS	TECHNIQUES	FREQUENCY
Weeks 1–4	Decompression sphenobasilar synchondrosis Release of mid-cervical fascia and visceral component Buccal floor release Craniosacral condylar decompression Release visceral component of the stomach Release of the diaphragm	Fronto-occipital approach in decompression Occiput-sternum fascial agreement technique Technique of inhibition of the buccal floor and direct inhibition of the mylohyoid mm Muscle inhibition technique combined with craniosacral condylar decompression Seated cardiac relaxation technique Functional technique of the diaphragm	Once per week for 45 min
Weeks 5–8	Occiput-mastoid suture release Tongue fascial release in relation to the hyoid bone Anterior cervical arch release Visceral component release in relation to the stomach	Fascial technique in relation to the temporal bone and occiput Tongue-structure fascial agreement technique Anterior cervical arch technique Relaxation of the cardia with a long lever	Once per week for 45 min

**Table 3:** Patient-reported outcome measure scores at the beginning and at the end of the study.

OUTCOME	PRE-TREATMENT (Before the 1st OMT Session)	POST-TREATMENT (After the 8th OMT Session)
Strand Scale and Swallowing tests	Slowed chewing movements and delayed gag reflex	Chewing movements suitable for chewing solid foods Slight tendency to fall pre-swallowing with liquid bolus taken with free oral cavity
DYALS	Difficulty swallowing liquid foods Cough when swallowing solid foods and liquids Need to drink in several sips	Cough present occasionally with fluid intake There is no need to drink in small sips
ALSFRS-R	45/48	45/48
ALSAQ-40	Swallowing: 41.6, sometimes problematic Communication: 53.5, sometimes problematic. Emotional aspect: 24, rarely problematic	Swallowing: 25, rarely problematic Communication: 59, problems sometimes Emotional aspect: 15, no problem

## Data Availability

No new data were created or analyzed in this study.
